# Associations of thyroid hormone serum levels with in-vivo Alzheimer’s disease pathologies

**DOI:** 10.1186/s13195-017-0291-5

**Published:** 2017-08-17

**Authors:** Hyo Jung Choi, Min Soo Byun, Dahyun Yi, Bo Kyung Sohn, Jun Ho Lee, Jun-Young Lee, Yu Kyung Kim, Dong Young Lee, for the KBASE Research Group

**Affiliations:** 1 Department of Neuropsychiatry, Seoul National University Boramae Medical Center, Seoul, South Korea; 2 Institute of Human Behavioral Medicine, Medical Research Center Seoul National University, Seoul, South Korea; 3 Department of Psychiatry, Inje University Sanggye Paik Hospital & Inje University College of Medicine, Seoul, South Korea; 4 Department of Neuropsychiatry, Seoul National University Hospital, Seoul, South Korea; 5 Department of Psychiatry, Seoul National University College of Medicine, 101 Daehak-ro, Jongno-gu, Seoul, 110-744 South Korea; 6 Department of Nuclear Medicine, Seoul National University Boramae Medical Center, Seoul, South Korea

**Keywords:** Beta-amyloid, Neurodegeneration, Thyroid hormone, Thyroid-stimulating hormone, Alzheimer’s disease, Biomarker

## Abstract

**Background:**

The present study investigated the relationships between thyroid hormone serum levels or thyroid-stimulating hormone (TSH) and two Alzheimer’s disease (AD)-specific biomarkers, cerebral amyloid beta (Aβ) burden and glucose metabolism, in AD-signature brain regions in cognitively normal (CN) middle-aged and older individuals.

**Methods:**

This study assessed 148 CN individuals who received comprehensive clinical and neuropsychological assessments that included ^11^C-Pittsburgh Compound B (PiB)-positron emission tomography (PET) scans, ^18^F-deoxyglucose (FDG)-PET scans, and the quantification of serum triiodothyronine (T3), free T3, free thyroxine (fT4), and TSH levels.

**Results:**

All participants were clinically euthyroid. Independent negative associations were found between serum fT4 levels and global cerebral Aβ deposition after controlling for the effects of age, gender, and the apolipoprotein E ε4 (*APOE*ε4) genotype. Although serum TSH levels were not associated with global cerebral Aβ deposition, they had a significant negative association with glucose metabolism in the precuneus/posterior cingulate cortex after controlling for age, gender, and the *APOE*ε4 genotype. No other thyroid hormones exhibited relationships with either brain Aβ burden or glucose metabolism.

**Conclusions:**

Even in a clinical euthyroid state, low serum fT4 and high serum TSH levels appear to be differentially associated with AD-specific brain changes.

**Electronic supplementary material:**

The online version of this article (doi:10.1186/s13195-017-0291-5) contains supplementary material, which is available to authorized users.

## Background

Several studies have identified an association between dysregulation of thyroid hormones and Alzheimer’s disease (AD) dementia [1–5]. However, whether serum levels of thyroid hormones are associated with AD pathologies, such as cerebral amyloid beta protein (Aβ) deposition and neurodegeneration, in the living human brain remains unclear.

Preclinical studies have repeatedly found an association between thyroid hormones and brain Aβ deposition in mice [6–9] and in human brain-derived neuroblastoma cells [9]. Additionally, two pathological studies of postmortem human brain tissues showed that thyroid hormone levels and Aβ deposition are related [5, 10]. Cerebral Aβ deposition begins 10–20 years before development of AD dementia [11] and reaches a state of near saturation in the stages of dementia or mild cognitive impairment [12, 13]. Consequently, detection of an association between thyroid hormone serum levels and cerebral Aβ deposition may be difficult in cognitively impaired individuals. Therefore, it is important to investigate the relationships of thyroid hormones in serum with in-vivo cerebral Aβ deposition in cognitively normal (CN) individuals. Furthermore, region-specific neurodegeneration is another important pathological change to consider in the AD brain. ^18^F-Deoxyglucose (FDG)-positron emission tomography (PET) has been used to measure regional cerebral glucose metabolism (rCMglu), and the specific pattern of rCMglu reduction in FDG-PET is regarded as a presymptomatic biomarker for AD [14]. However, few studies have investigated the association between thyroid hormone serum levels and the AD-specific rCMglu pattern.

Therefore, this study investigated the relationships of serum levels of thyroid hormones with in-vivo AD neuropathologies, including cerebral Aβ burden and neurodegeneration, in AD-signature regions in CN middle-aged and older individuals.

## Methods

### Participants

This study was part of the Korean Brain Aging Study for Early Diagnosis and Prediction of Alzheimer’s Disease (KBASE), which is an ongoing prospective cohort study searching for new biomarkers of AD with the aim of identifying the associations of various lifetime experiences with AD-related brain changes. The present study assessed 148 CN middle-aged and older subjects. The inclusion criteria were: aged between 55 and 90 years (inclusive); a Clinical Dementia Rating score [15] of 0; and no diagnosis of mild cognitive impairment or dementia. The exclusion criteria were: any present serious medical, psychiatric, or neurological disorders that could affect mental function; the presence of severe communication problems that would make a clinical examination or brain scans difficult; contraindications for magnetic resonance imaging (MRI) scans (e.g., pacemaker, claustrophobia, etc.); the absence of a reliable informant; illiteracy; and participation in another clinical trial and/or treatment with an investigational product.

The Institutional Review Board of the Seoul National University Hospital and SNU-SMG Boramae Center, South Korea, approved the study and all participants provided written informed consent.

### Clinical assessment

All participants completed standardized clinical assessments administered by trained psychiatrists that were based on the KBASE clinical assessment protocol, which incorporates the Korean version of the Consortium to Establish a Registry for Alzheimer’s Disease Assessment Packet (CERAD-K) [16]. Additionally, the KBASE neuropsychological assessment protocol, which incorporates the CERAD neuropsychological battery [17], was administered to all participants by trained neuropsychologists. The presence or absence of stroke, diabetes, and hyperlipidemia and histories of transient ischemic attack (TIA), hypertension, and coronary artery disease were assessed systematically to create a composite score for vascular risk; this score was the sum of the factors (if present) and ranged from 0 to 6 [18].

### Laboratory tests of blood samples

Blood samples were obtained via venipuncture after an overnight fast. Serum levels of total triiodothyronine (T3), free T3 (fT3), free thyroxine (fT4), and thyroid-stimulating hormone (TSH) were evaluated with a chemiluminescence immunoassay using the ADVIA Centaur XP system (Siemens, Washington, DC, USA). The normal range for total T3 is 65–150 ng/dl, for fT3 is 2.3–4.2 pg/ml, for fT4 is 0.89–1.76 ng/dl, and for TSH is 0.55–4.78 μIU/ml; serum TSH and fT4 levels were assessed to define thyroid status. Additionally, genomic DNA was extracted from whole blood samples to perform apolipoprotein E (*APOE*) genotyping, as described previously [19]. Participants with at least one APOE ε4 allele (*APOE*ε4) were identified as *APOE*ε4 carriers.

### ^11^C-Pittsburgh Compound B-PET image acquisition and preprocessing

All participants underwent simultaneous three-dimensional ^11^C-Pittsburgh Compound B (PiB)-PET and 3D T1-weighted MRI scans with a 3.0 T Biograph mMR (PET-MR) scanner (Siemens) according to the manufacturer’s approved guidelines. Prior to the scan, each participant received an intravenous administration of 555 MBq of PiB (range 450–610 MBq) and then rested in a waiting room for 40 min.

PiB-PET data collected in list mode were processed for routine corrections such as uniformity, ultrashort echo time (UTE)-based attenuation, and decay corrections and were then reconstructed into a 256 × 256 image matrix using iterative methods (six iterations with 21 subsets). T1-weighted images were acquired in the sagittal orientation using the following characteristics: repetition time = 1670 ms, echo time = 1.89 ms, field of view = 250 mm, 256 × 256 matrix, and slice thickness = 1.0 mm. Additionally, fluid-attenuated inversion recovery (FLAIR) and T2-weighted images were obtained for qualitative clinical readings.

All image preprocessing steps were performed using Statistical Parametric Mapping 8 (SPM8) implemented in Matlab 2014a (Mathworks, Natick, MA, USA). Static PiB-PET images were coregistered to an individual T1 structural image and then the transformation parameters for the spatial normalization of the individual T1 image to a standard Montreal Neurological Institute (MNI) template were calculated. Using IBASPM software, inverse transformation parameters were used to bring the Automated Anatomical Labeling (AAL) 116 atlas [20] in a standard space to an individual space for each subject (resampling voxel size = 1 mm × 0.98 mm × 0.98 mm); the nongray matter portions of the atlas were individually masked using the cerebral gray matter segment image of each subject. Using the individual AAL116 atlas, the mean regional PiB uptake values from cerebral regions were extracted from the T1-coregistered PiB-PET images. The cerebellar gray matter was used as the reference region for the quantitative normalization of cerebral PiB uptake values due to its relatively low Aβ deposition [21]. To measure PiB uptake in the cerebellar gray matter regions, a probabilistic cerebellar atlas (Institute of Cognitive Neuroscience, UCL, UK; Cognitive Neuroscience Laboratory, Royal Holloway, UK) was brought into individual space in the same manner as already described. Of the 28 anatomical structural regions in the cerebellar atlas, the cerebellar lobular regions (except for the vermis) were included to extract the mean cerebellar uptake values.

The AAL algorithm and a region combining method [22] were applied to set regions of interest (ROIs) to characterize PiB retention levels in the frontal, lateral parietal, precuneus/posterior cingulate cortex (PCC), and lateral temporal regions, where prominent PiB retention has been reported [23]. Standardized uptake value ratio (SUVR) values for each ROI were calculated by dividing the mean value of all voxels within each ROI by the mean cerebellar uptake value in the same image. Additionally, a global cortical ROI consisting of the four ROIs was defined and a global cortical SUVR was generated by dividing the mean value of all voxels of the global cortical ROI by the mean cerebellar uptake value in the same image. Global cerebral Aβ deposition was defined as the mean PiB retention value of the global cortical ROI. Images were classified as amyloid-positive if the mean ^11^C-PiB retention value was over 1.4 in at least one of the following ROIs: frontal, lateral temporal, lateral parietal, or posterior cingulate-precuneus (PC-PRC) [22].

### FDG-PET image acquisition and preprocessing

The participants also underwent FDG-PET scans using the same PET-MR machine described earlier. Prior to the scan, each participant fasted for at least 6 h, received an intravenous administration of FDG radioligands (0.1 mCi/kg), and then rested in a waiting room for 40 min. PET data collected in list mode (5 min × four frames) were processed for routine corrections such as uniformity, UTE-based attenuation, and decay corrections. Following an inspection for any significant head movements, the data were reconstructed into a 20-min summed image using iterative methods (six iterations with 21 subsets).

The following image processing steps were performed using SPM12 implemented in Matlab 2014a (Mathworks). Static FDG-PET images were coregistered to an individual T1 structural image and then the transformation parameters for the spatial normalization of the individual T1 image to a standard MNI template were calculated for the utilization of the spatial normalization of FDG-PET images to a standard MNI space. After smoothing the spatially normalized FDG-PET images with a 12-mm Gaussian filter, intensity normalization was performed using the pons as a reference region. SUVR values were extracted from regions known to be sensitive to changes associated with AD [24–26], including the angular gyri, PCC, precuneus, and inferior temporal gyri. The AD-signature region CMglu was defined as the weighted mean of the four ROIs.

### Statistical analyses

In order to examine the relationships of serum levels of thyroid hormones or TSH with cerebral Aβ deposition or CMglu, we took two steps of statistical analyses. Before analysis, global cerebral Aβ deposition was natural log-transformed to reduce the skewness that existed in the distributions. In the first step, we conducted Pearson correlation analyses to explore the associations between hormones and imaging variables including global cerebral Aβ deposition and AD-signature region CMglu. Based on the results from preliminary exploratory analyses, variables with *p* < 0.1 were selected for the second-step multivariate analyses. In the second step, we tested the multiple linear regression model(s) with the hormone selected from the first step as an independent variable and the corresponding imaging marker as a dependent variable controlling age, gender, and *APOE ε4* genotype as covariates. For global cerebral Aβ deposition, we conducted the same regression analyses controlling VRS as an additional covariate. For CMglu, we conducted the same regression analyses controlling global cerebral Aβ deposition or VRS as an additional covariate. For the second step of the analyses, we applied strict threshold by applying Bonferroni correction for multiple testing (*p* < 0.05/2 (number of associations selected for the second step of analyses) = 0.025). All of the statistical tests were conducted using the Statistical Package for the Social Sciences for Windows version 20.0 (SPSS Inc., Chicago, IL, USA).

## Results

### Demographic and clinical characteristics

The demographic and clinical characteristics of the study participants (*n* = 148) are summarized in Table 1. The global cerebral Aβ deposition SUVR was 1.16 ± 0.23 (range 0.56–2.54). The proportion of amyloid positive subjects were 10.8% (*n* = 16). All participants were clinically euthyroid but subclinical thyroid problems were found in 12 individuals (8.1%); of these 12 participants, nine had high TSH levels and three had low TSH levels.

**Table 1:** Demographic and clinical characteristics

	CN (*n* = 148)
Age (years)	68.93 ± 7.85
Education (years)	11.67 ± 4.85
Gender, female (%)	92 (62.2)
CDR	0
*APOE ε4* allele(+) (%)	25 (16.9)
HRSD	0.86 ± 1.60
VRS	1.07 ± 0.92
Neuropsychological tests^a^	
MMSE score	26.94 ± 2.58
Animal fluency	16.22 ± 4.70
Boston naming	12.27 ± 2.37
Word list learning	20.15 ± 4.15
Constructional praxis	10.06 ± 1.37
Word list recall	6.76 ± 1.83
Word list recognition	9.24 ± 1.04
Constructional recall	7.46 ± 2.80
Global amyloid burden (SUVR)	1.16 ± 0.23
Amyloid positivity (%)	16 (10.8)
T3 (mg/dl)	103.84 ± 17.34
Free T3 (pg/ml)	3.11 ± 0.34
Free T4 (ng/dl)	1.17 ± 0.16
TSH (μIU/ml)	2.36 ± 1.57

Data for continuous variables presented as a mean ± SD. Categorical variables presented as *N* (%)

*APOE* apolipoprotein E, *CDR* Clinical Dementia Rating, *CN* cognitively normal, *HRSD* Hamilton Depression Rating Score, *VRS* vascular risk score, *MMSE* Mini-mental State Examination, *SUVR* standardized uptake value ratio, *T3* triiodothyronine, *T4* thyroxine, *TSH* thyroid-stimulating hormone

^a^ *n* = 147

### Exploratory univariate analyses

In the exploratory step of the analyses using Pearson’s correlation, we found that the associations between serum fT4 and global cerebral Aβ deposition, as well as between TSH and CMglu in the AD-signature region, are below the threshold (*p* < 0.1) (Additional file 1: Table S1). Based on the results, we selected these associations for the second confirmatory step of the analyses.

### Confirmatory multivariate analysis for global cerebral Aβ deposition

**Fig. 1:**
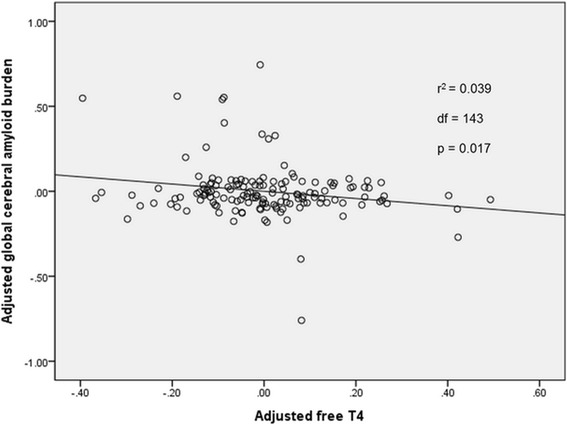
Partial regression plot showing the relationship between serum fT4 and cerebral Aβ in CN participants. Illustration of the partial regression model predicting natural log-transformed cerebral Aβ according to serum fT4 values. Control variables included age, gender, and the *APOE ε4* genotype. *fT4* free thyroxine, *Aβ* global cerebral amyloid burden, *APOE* apolipoprotein E, *CN* cognitively normal

Based on the results from the first step, we selected serum fT4 as the candidate hormone for further analyses for global cerebral Aβ deposition. Multiple linear regression analyses controlling for age, gender, and *APOE ε4* carrier status revealed that global cerebral Aβ deposition had a significant negative association with serum fT4 levels (Table 2, Fig. 1). Serum fT4 explained 3.9% of the variance of global cerebral Aβ deposition. Controlling VRS in addition to age, gender, and *APOEε4* status did not largely change the results (Table 2). Similarly, excluding the participants (*n* = 5) who took medications with the potential to affect thyroid function (e.g., synthroid, propylthiouracil, and methimazole) did not change the results (Additional file 2: Table S2).

**Table 2:** Multiple linear regression analysis with global cerebral Aβ deposition as the dependent variable (*n* = 148)

Dependent variable	Independent variable	Model I^a^				Model II^b^			
		*B*	SE	*t*	*p*	*B*	SE	*t*	*p*
Global cerebral Aβ deposition	Serum fT4 level	– 0.213	0.088	– 2.407	0.017*	– 0.205	0.088	– 2.316	0.022*

Multiple linear regression analysis was conducted to investigate the relationship between serum fT4 level and global cerebral Aβ deposition controlling for several variables. Global Aβ deposition values were natural log-transformed to normalize variance

*Aβ* amyloid beta protein, *APOE* apolipoprotein E, *B* regression coefficient, *SE* standard error, *fT4* free thyroxine

*p < 0.025 (statistically significant)

^a^Adjusted for age, gender, and *APOE* ε4 carrier status

^b^Adjusted for age, gender, *APOE* ε4 carrier status, and vascular risk score

To explore whether or not one particular ROI is driving the relationship for global cerebral Aβ deposition and serum fT4, the associations between each regional cerebral Aβ and serum fT4 were also examined using multiple regression analysis controlling for age, gender, and *APOEε4* status. Serum fT4 showed significant associations with frontal, lateral temporal, and lateral parietal regional cerebral Aβ deposition and a trend-level association with PC-PRC regional cerebral Aβ deposition (Additional file 3: Table S3), indicating no regional predominance of the relationship.

**Fig. 2:**
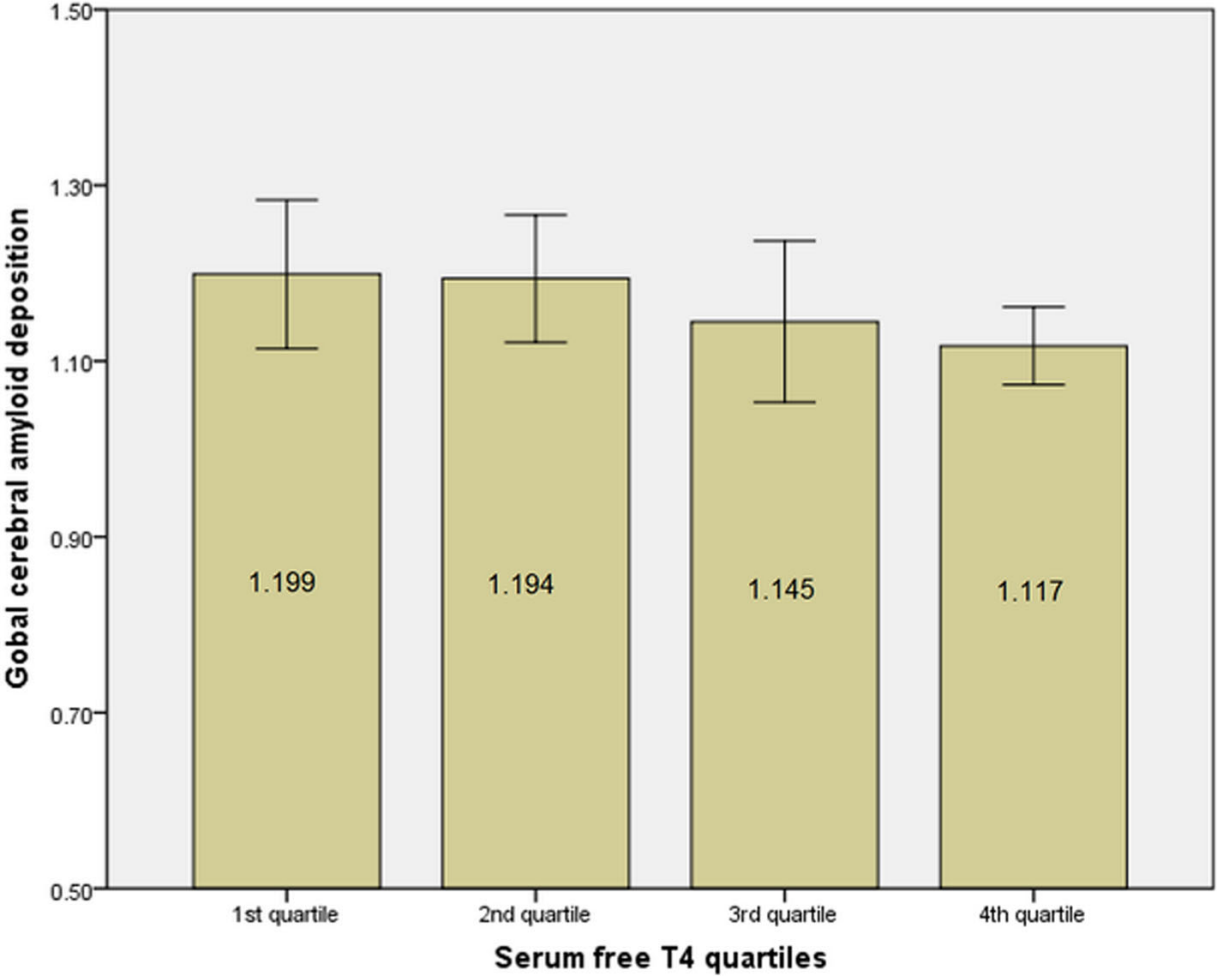
Global cerebral Aβ deposition according to the quartiles of fT4 levels in the study population. When subjects were divided according to category of fT4 (quartiles of similar sizes), higher levels of free T4 showed lower global cerebral amyloid deposition. Values presented as mean and *error bars* represent standard error. *fT4* free thyroxine, *Aβ* global cerebral amyloid burden

Additionally, in order to explore the clinically meaningful serum fT4 level, we divided the fT4 level into four quartiles and compared the global amyloid deposition between the quartiles using general linear model analyses. Although statistically not significant, there was a trend of negative association between quartiles of serum fT4 concentrations and mean global cerebral Aβ deposition (Additional file 4: Table S4). Participants with the lowest quartile of fT4 concentration had a mean SUVR of 1.199, whereas those in the highest quartile of fT4 concentration had a mean SUVR of 1.117 (Fig. 2).

### Confirmatory multivariate analysis for CMglu

Based on the results of univariate analyses, we selected serum TSH as the candidate hormone for further multivariate analysis for CMglu in the AD-signature regions. However, multiple linear regression analyses controlling for age, gender, and APOE ε4 carrier status showed that serum TSH levels were not related to CMglu in the AD-signature regions (Table 3). The addition of global cerebral Aβ deposition and VRS as covariates did not largely change the results. Additional exploratory multiple regression analyses for the relationship between serum TSH and each regional CMglu showed that the serum TSH level had significant association with CMglu in the precuneus, but did not have any association with CMglu of the other regions (Additional file 5: Table S5).

**Table 3:** Multiple linear regression analysis with AD-signature CMglu as a dependent variable (*n* = 148)

Model I^a^					Model II^b^				Model III^c^			
*B*		SE	*t*	*p*	*B*	SE	*t*	*p*	*B*	SE	*t*	*p*
AD-signature region CMglu												
TSH	– 0.011	0.006	– 1.677	0.096	– 0.011	0.006	– 1.667	0.098	– 0.010	0.007	– 1.486	0.139

Multiple linear regression analysis was conducted to investigate the relationship between serum TSH and AD-signature CMglu controlling for several variables

*B* regression coefficient, *SE* standard error, *CMglu* cerebral glucose metabolism, *APOE* apolipoprotein E, *TSH* thyroid-stimulating hormone, *AD* Alzheimer’s disease, *Aβ* amyloid beta protein

^a^Adjusted for age, gender, and *APOE* ε4 carrier status

^b^Adjusted for age, gender, *APOE* ε4 carrier status, and global Aβ retention (natural log-transformed)

^c^Adjusted for age, gender, *APOE* ε4 carrier status, global Aβ retention (natural log-transformed), and vascular risk score

## Discussion

The present study examined the relationships between serum levels of thyroid hormones or TSH and AD-specific brain biomarkers (i.e., an amyloid biomarker and a neurodegeneration biomarker) in CN middle-aged and older individuals with no clinical symptoms of thyroid disease. Low serum fT4 levels were associated with an increase in cerebral Aβ deposition, and fT4 explained 3.9% of the variance of global cerebral Aβ deposition. Although statistically not significant, there was a trend of negative association between quartiles of serum fT4 concentrations and mean global cerebral Aβ deposition. Given that the mean global cerebral Aβ deposition level largely decreased from the second quartile to the third quartile of the free T4 level (Fig. 2, Additional file 5: Table S5), individuals with a serum fT4 level < 1.165 ng/dl (quartile 3) appear relatively more vulnerable to cerebral Aβ deposition than those with a higher fT4 level. To the best of our knowledge, this is the first report to reveal associations between serum thyroid hormones and cerebral Aβ burden and AD-specific neurodegeneration in euthyroid CN older individuals.

The mechanisms underlying the relationship between serum levels of fT4 and cerebral Aβ deposition are not yet fully understood. Serum fT4 crosses the blood–brain barrier (BBB) via monocarboxylate transporter 8 (MCT 8) and reaches the astrocytes where it is converted to T3 by type 2 deiodinase (D2) [27]. Brain T3 can suppress the cerebral gene expression of beta-amyloid precursor protein (APP) [9]. In the present study, serum fT3 was not associated with brain amyloid burden, which may have been due to its small contribution to brain T3. In the cerebral cortex, active T3 is predominantly derived from serum T4 rather than serum T3 [28] because serum T3 seems to be degraded by tyrosyl ring deiodinase before it reaches the neuronal space [29].

Preclinical studies have found a negative association between brain T3 and APP expression using a transgenic mouse model of AD [8, 9, 30], and a human autopsy study [10] revealed decreases in cerebral T3 levels in subjects at Braak stages IV–V, which is similar to the present results. In contrast to the present results, a postmortem human study [5] reported that higher serum levels of total T4 but not fT4 are associated with an increase in neocortical neuritic plaques. Serum levels of total T4 can be affected by the concentration of thyroid hormone-binding proteins, which fluctuate due to various medical conditions. Moreover, this autopsy study assessed the brain tissues of AD dementia patients in which the Aβ deposition may have already been saturated, which would make it difficult to identify an association between serum fT4 levels and neocortical amyloid burden. On the other hand, the present study included only CN older individuals far from Aβ saturation.

Although serum TSH levels were not significantly associated with overall metabolism in AD-signature regions, exploratory analysis indicated that it may be negatively associated with CMglu in the precuneus, where AD-related hypometabolism first occurs [27–29]. This is partially consistent with the findings of a previous study [31] showing that serum TSH levels are negatively associated with global CMglu in euthyroid mood disorder patients. The mechanisms linking serum TSH with CMglu are not well understood. It is possible that serum dyslipidemia mediates elevations in TSH and decreases in CMglu because subclinical hypothyroidism may lead to elevated serum levels of total cholesterol [32], which are associated with lower CMglu in various brain regions, including the precuneus, during late middle age [33].

The present study has several limitations. First, this is a cross-sectional study and, therefore, it is difficult to identify causal relationships based on these findings. Further longitudinal studies are needed to determine the nature of the associations between serum thyroid hormones or TSH and brain amyloid burden. Additionally, the lack of repeated assessments of thyroid hormone levels might have resulted in some errors in measurements of the serum levels because there are diurnal/seasonal variations in thyroid hormone levels. However, in order to minimize such errors, thyroid hormones were assessed at the same time (9–10 a.m.) in all participants.

## Conclusions

The present results suggest that, even in a clinical euthyroid state, low serum fT4 and high serum TSH levels are differentially associated with AD-related brain changes (i.e., increases in global cerebral amyloid burden and increases in precuneus hypometabolism, respectively). Further longitudinal studies are needed to clarify whether stricter correction of serum thyroid hormone levels will be helpful for attenuating AD specific brain pathologies.

### Additional files

Additional file 1: Table S1. Presenting association between serum thyroid hormone or TSH and global cerebral Aβ deposition or CMglu. Pearson correlation analysis was performed to investigate the relationship between serum thyroid hormones or TSH and global cerebral Aβ deposition or CMglu. Global cerebral Aβ values were natural log-transformed to normalize variance. (DOCX 16 kb)

Additional file 2: Table S2. Presenting multiple linear regression analysis for participants without thyroid medication (*n* = 143). Multiple linear regression analysis was performed to investigate the relationship between serum fT4 level and global cerebral Aβ deposition after controlling several variables. Global cerebral Aβ deposition values were natural log-transformed to normalize variance. (DOCX 16 kb)

Additional file 3: Table S3. Presenting multiple regression analyses with regional cerebral Aβ deposition as the dependent variable. Multiple linear regression analysis was performed to investigate the relationship between serum fT4 and regional cerebral Aβ deposition after controlling age, gender, and *APOE* ε4 genotype (df for *F* statistics = 4143). All regional Aβ deposition values were natural log-transformed to normalize variance. (DOCX 19 kb)

Additional file 4: Table S4. Presenting global cerebral Aβ deposition by categories of fT4 serum level. To compare global cerebral Aβ deposition by quartiles of fT4 serum level, general linear models were used with adjustment for age, gender, and *APOE* ε4 genotype. (DOCX 16 kb)

Additional file 5: Table S5. Presenting multiple regression analyses with rCMglu as the dependent variable. Multiple linear regression analysis was performed to investigate the relationship between serum TSH and rCMglu after controlling for age, gender, and *APOE* ε4 genotype (df for *F* statistics = 4143). (DOCX 18 kb)

## Appendix

### KBASE Research Group

Dong Young Lee, MD, PhD (Seoul National University, Principal Investigator); Min Soo Byun, MD, PhD (Seoul National University, Core PI Clinical & Executive); Dahyun Yi, PhD (Seoul National University, Core PI Neuropsychology); Yu Kyeong Kim, MD, PhD (SMG-SNU Boramae Medical Center, Core PI PET); Chul-Ho Sohn, MD, PhD (Seoul National University, Core PI MRI); Inhee Mook-Jung, PhD (Seoul National University, Core PI Biomarker); Murim Choi, PhD (Seoul National University, Core PI Genetics); Yu Jin Lee, MD, PhD (Seoul National University, Core PI Sleep), Seokyung Hahn, PhD (Seoul National University, Core PI Biostatistics); Hyun Jung Kim, MD (Changsan Convalescent Hospital, coinvestigator); Mun Young Chang, MD (Chung-Ang University College of Medicine, coinvestigator); Seung Hoon Lee, MD (Daerim St. Mary’s Hospital, coinvestigator); Jee Wook Kim, MD, PhD (Hallym University Dongtan Sacred Heart Hospital, coinvestigator); Jong-Min Lee, PhD (Hanyang University, coinvestigator); Dong Woo Lee, MD, PhD (Inje University Snaggye Paik Hospital, coinvestigator); Bo Kyung Sohn, MD (Inje University Snaggye Paik Hospital, coinvestigator); Seok Woo Moon, MD, PhD (Konkuk University Chungju Hospital, coinvestigator); Man Ho Choi, PhD (Korea Institute of Science and Technology, coinvestigator); Sang-Won Lee, PhD (Korea University, coinvestigator); Hyewon Baek, MD (Kyunggi Provincial Hospital for the Elderly, coinvestigator); Na Young Han, MD (National Research Center for Dementia, coinvestigator); Jong-Won Kim, MD, PhD (Samsung Medical Center, coinvestigator); Seung-Ho Ryu, MD, PhD (School of Medicine Konkuk University, coinvestigator); Shin Gyeom Kim, MD, PhD (Soonchunhyang University Hospital Bucheon, coinvestigator); Sun-Ho Han, PhD (Seoul National University, coinvestigator); Jae Sung Lee, PhD (Seoul National University, coinvestigator); Yun-Sang Lee, PhD (Seoul National University, coinvestigator); Jong Inn Woo, MD, PhD (Seoul National University, coinvestigator); Sang Eun Kim, MD, PhD (Seoul National University Bundang Hospital, coinvestigator); Byung Chul Lee, PhD (Seoul National University Bundang Hospital, coinvestigator); Gi Jeong Cheon, MD, PhD (Seoul National University Hospital, coinvestigator); Koung Mi Kang, MD (Seoul National University Hospital, coinvestigator); Jee-Eun Park, MD, PhD (Seoul National University Hospital, coinvestigator); Hyeong Gon Yu, MD, PhD (Seoul National University Hospital, coinvestigator); Jun-Young Lee, MD, PhD (SMG-SNU Boramae Medical Center, coinvestigator); Hyo Jung Choi, MD (SMG-SNU Boramae Medical Center, coinvestigator); Young Min Choe, MD (University of Ulsan College of Medicine, Ulsan University Hospital, coinvestigator); Woonhyung Ghim, MD (Seoul National University Hospital, research fellow); So Yeon Jeon, MD (Seoul National University Hospital, research fellow); Woo Jin Kim, MD, PhD (Seoul National University Hospital, research fellow); Kang Ko, MD (Seoul National University Hospital, research fellow); Jun Ho Lee, MD (Seoul National University Hospital, research fellow); Kyoungjin Chu (Seoul National University Hospital, psychologist); Hyunwoong Ko (Seoul National University Hospital, psychologist); Younghwa Lee (Seoul National University Hospital, psychologist); Donghwi Hwang (Seoul National University, image analyst); Seugn Kwan Kang (Seoul National University, image analyst); Seong A Shin (Seoul National University, image analyst); Jeong Yeon Hwang, MD (Seoul National University, data analyst); Jong-Chan Park (Seoul National University, data analyst); Jong-Ho Park (Samsung Medical Center, genetic data analyst); Jieun Seo (Seoul National University, genetic data analyst); Mi Ae Han (Seoul National University Hospital, research coordinator); Eun A Jo (Seoul National University Hospital, research coordinator); Gi Jung Jung (Seoul National University Hospital, research coordinator); Jin Hee Keum (Seoul National University Hospital, research coordinator); Mi Sun Kim (SMG-SNU Boramae Medical Center, research coordinator); Min Jeong Kim (Seoul National University Hospital, research coordinator); Han Na Lee (Seoul National University Hospital, research coordinator); Bo Eun Park (Seoul National University Hospital, research coordinator); Ji Sun Shin (Seoul National University Hospital, research coordinator); Yun Jung Hwang (Seoul National University Hospital, researcher); Joon Hyung Jung, MD (Seoul National University Hospital, researcher); Kiyoung Sung, MD (Seoul National University Hospital, researcher); Eun Hye Kim (Seoul National University, research assistant); and Han Byul Choi (National Research Center for Dementia, administrative staff).

### KBASE Partner Organizations

Dongjak-gu Center for Dementia; Jongno-gu Center for Dementia; Ministry of Science, ICT and Future planning; National Research Center for Dementia; National Research Foundation of Korea; Seoul Metropolitan Center for Dementia; Seoul National University; Seoul National University Bundang Hospital; Seoul National University Hospital; SMG-SNU Boramae Medical Center; and The Korean Association for Dementia.

## Abbreviations

AAL: Automated Anatomical Labeling
AD: Alzheimer’s disease
*APOE*: Apolipoprotein E
APP: Beta-amyloid precursor protein
Aβ: Amyloid beta
BBB: Blood–brain barrier
CERAD-K: Consortium to Establish a Registry for Alzheimer’s Disease Assessment Packet
CMglu: Cerebral glucose metabolism
CN: Cognitively normal
FDG: ^8^F-Deoxyglucose
FLAIR: Fluid-attenuated inversion recovery
fT3: Free T3
fT4: Free thyroxine
KBASE: Korean Brain Aging Study for Early Diagnosis and Prediction of Alzheimer’s Disease
MNI: Montreal Neurological Institute
MRI: Magnetic resonance imaging
PCC: Posterior cingulate cortex
PET: Positron emission tomography
PiB: ^11^C-Pittsburgh Compound B
ROI: Region of interest
SPM8: Statistical Parametric Mapping 8
SUVR: Standardized uptake value ratio
T3: Total triiodothyronine
TIA: Transient ischemic attack
TSH: Thyroid-stimulating hormone
UTE: Ultrashort echo time
VRS: Vascular risk score
