# Supplementary material for: Associations of thyroid hormone serum levels with in-vivo Alzheimer’s disease pathologies
Source: Alzheimers Res Ther. 2017 Aug 17;9:64. doi: 10.1186/s13195-017-0291-5 (PMC5561599; doi:10.1186/s13195-017-0291-5)
Supplement: Supplementary file 1 — Presenting association between serum thyroid hormone or TSH and global cerebral Aβ deposition or CMglu. Pearson correlation analysis was performed to investigate the relationship between serum thyroid hormones or TSH and global cerebral Aβ deposition or CMglu. Global cerebral Aβ values were natural log-transformed to normalize variance. (DOCX 16 kb) [file 13195_2017_291_MOESM1_ESM.docx]

| **Table S1. Relationship between serum thyroid hormones or TSH levels and global cerebral Aβ deposition or CMglu** | | |
| --- | --- | --- |
|  | Correlation coefficients (*p*-value) | |
|  | global cerebral Aβ deposition | CMglu in AD-signature region |
| Free T4 | -.156 (0.059)* | 0.091 (0.273) |
| Free T3 | .031 (0.712) | 0.050 (0.542) |
| T3 | .131 (0.114) | 0.049 (0.553) |
| TSH | .054 (0.517) | -0.176 (0.033)* |
| Pearson correlation analysis was done for investigating the relationship between serum thyroid hormones or TSH and global cerebral Aβ deposition or CMglu (df = 146). Global cerebral Aß values were natural log-transformed to normalize variance. *p<0.1 ; Abbreviations: Aß, amyloid beta protein; CMglu, cerebral glucose metabolism; PCC, posterior cingulate cortex; AD, Alzhiemer’s disease; T4, thyroxine; T3, triiodothyronine; TSH, thyroid-stimulating hormone | | |
