# Supplementary material for: Associations of thyroid hormone serum levels with in-vivo Alzheimer’s disease pathologies
Source: Alzheimers Res Ther. 2017 Aug 17;9:64. doi: 10.1186/s13195-017-0291-5 (PMC5561599; doi:10.1186/s13195-017-0291-5)
Supplement: Supplementary file 2 — Presenting multiple linear regression analysis for participants without thyroid medication (n = 143). Multiple linear regression analysis was performed to investigate the relationship between serum fT4 level and global cerebral Aβ deposition after controlling several variables. Global cerebral Aβ deposition values were natural log-transformed to normalize variance. (DOCX 16 kb) [file 13195_2017_291_MOESM2_ESM.docx]

| **Table S2. Multiple linear regression analysis for participants without thyroid medication (n = 143)** | | | | | | | | | |
| --- | --- | --- | --- | --- | --- | --- | --- | --- | --- |
| Dependent variable | Independent variable | **Model I**^a^ | | | | **Model II**^b^ | | | |
|  |  | B | SE | t | *p* | B | SE | t | *p* |
| Global cerebral Aß deposition |  |  |  |  |  |  |  |  |  |
|  | Serum fT4 level | -.221 | .092 | -2.396 | **.018*** | -.210 | .092 | -2.274 | **.024*** |
| Multiple linear regression analysis was done for investigating the relationship between serum fT4 level and global cerebral Aß deposition after controlling several variables. Global cerebral Aß deposition values were natural log-transformed to normalize variance.;*p < 0.025 ^a^Model I: Adjusted for age, gender, and *APOE* ε4 carrier status; ^b^Model II: Adjusted for age, gender, *APOE* ε4 carrier status and vascular risk score. Abbreviations: Aß, amyloid beta protein; *APOE*, apolipoprotein E; B, Regression coefficient; SE, Standard error; fT4, free thyroxine; | | | | | | | | | |
