# Supplementary material for: Associations of thyroid hormone serum levels with in-vivo Alzheimer’s disease pathologies
Source: Alzheimers Res Ther. 2017 Aug 17;9:64. doi: 10.1186/s13195-017-0291-5 (PMC5561599; doi:10.1186/s13195-017-0291-5)
Supplement: Supplementary file 3 — Presenting multiple regression analyses with regional cerebral Aβ deposition as the dependent variable. Multiple linear regression analysis was performed to investigate the relationship between serum fT4 and regional cerebral Aβ deposition after controlling age, gender, and APOE ε4 genotype (df for F statistics = 4143). All regional Aβ deposition values were natural log-transformed to normalize variance. (DOCX 19 kb) [file 13195_2017_291_MOESM3_ESM.docx]

| **Table S3. Multiple regression analyses with regional cerebral Aβ deposition as dependent variable** | | | | | | | | |
| --- | --- | --- | --- | --- | --- | --- | --- | --- |
| Covariates | B | SE | *t* | | | *p*-value | F (df) | R^2^ |
| Dependent variable: Aß Frontal region (df = 143) | | | | | | .025 | 2.866 (df = 4,143) | .048 |
| Age (years) | .002 | .002 | 1.275 | | | .204 |  |  |
| Sex | .041 | .030 | 1.342 | | | .182 |  |  |
| *APOE* ε4 genotype | .061 | .036 | 1.713 | | | .089 |  |  |
| Serum fT4 level | -.214 | .090 | -2.374 | | | .019 |  |  |
| Dependent variable: Aß Lateral temporal region (df = 143) | | | | | | .047 | 2.470 (df = 4,143) | .038 |
| Age (years) | .001 | .002 | | .840 | | .402 |  |  |
| Sex | .059 | .029 | | 2.055 | | .042 |  |  |
| *APOE* ε4 genotype | .024 | .034 | | .712 | | .478 |  |  |
| Serum fT4 level | -.209 | .085 | | -2.474 | | .015 |  |  |
| Dependent variable: Aß Lateral parietal region (df = 143) | | | | | | .038 | 2.613 (df = 4,143) | .042 |
| Age (years) | .002 | .002 | | 1.293 | | .198 |  |  |
| Sex | .050 | .031 | | 1.590 | | .114 |  |  |
| *APOE* ε4 genotype | .046 | .037 | | 1.235 | | .219 |  |  |
| Serum fT4 level | -.218 | .093 | | -2.338 | | .021 |  |  |
| Dependent variable : Aß PC/PRC region (df = 143) | | | | | | .043 | 2.530 (df = 4,143) | .040 |
| Age (years) | .002 | .002 | | | 1.205 | .230 |  |  |
| Sex | .064 | .035 | | | 1.810 | .072 |  |  |
| *APOE* ε4 genotype | .060 | .041 | | | 1.454 | .148 |  |  |
| Serum fT4 level | -.205 | .104 | | | -1.968 | .051 |  |  |
| Multiple linear regression analysis was done for investigating the relationship between serum fT4 and regional cerebral Aβ deposition after controlling age, gender, and *APOE* ε4 genotype (df for F statistics = 4,143). All regional Aß deposition values were natural log-transformed to normalize variance. Abbreviations: Aß, amyloid beta protein; B, regression coefficient; SE, standard error; *APOE*, apolipoprotein E; PC-PRC, posterior cingulate-precuneus; fT4, free thyroxine; | | | | | | | | |
