# Supplementary material for: Associations of thyroid hormone serum levels with in-vivo Alzheimer’s disease pathologies
Source: Alzheimers Res Ther. 2017 Aug 17;9:64. doi: 10.1186/s13195-017-0291-5 (PMC5561599; doi:10.1186/s13195-017-0291-5)
Supplement: Supplementary file 4 — Presenting global cerebral Aβ deposition by categories of fT4 serum level. To compare global cerebral Aβ deposition by quartiles of fT4 serum level, general linear models were used with adjustment for age, gender, and APOE ε4 genotype. (DOCX 16 kb) [file 13195_2017_291_MOESM4_ESM.docx]

| **Table S4. Global cerebral Aβ deposition by categories of fT4 serum level** | | | | | | |
| --- | --- | --- | --- | --- | --- | --- |
|  | Q1  (n = 38) | Q2  (n = 36) | Q3  (n = 37) | Q4  (n = 37) | F | p |
| Global cerebral Aβ  deposition (SUVR) | 1.20 ± 0.26 | 1.19 ± 0.22 | 1.14 ± 0.28 | 1.12 ± 0.13 | 2.127 | 0.099 |
| Data are shown as mean ± SD. To compare global cerebral Aβ deposition by quartiles of fT4 serum level, general linear models were used with adjustment for age, gender and *APOE* ε4 genotype. Abbreviation: Q, quartiles; Aβ, amyloid; SD, standard deviation; fT4, free thyroxine | | | | | | |
