# Supplementary material for: Associations of thyroid hormone serum levels with in-vivo Alzheimer’s disease pathologies
Source: Alzheimers Res Ther. 2017 Aug 17;9:64. doi: 10.1186/s13195-017-0291-5 (PMC5561599; doi:10.1186/s13195-017-0291-5)
Supplement: Supplementary file 5 — Presenting multiple regression analyses with rCMglu as the dependent variable. Multiple linear regression analysis was performed to investigate the relationship between serum TSH and rCMglu after controlling for age, gender, and APOE ε4 genotype (df for F statistics = 4143). (DOCX 18 kb) [file 13195_2017_291_MOESM5_ESM.docx]

| **Table S5. Multiple regression analyses with rCMglu as dependent variable** | | | | | | | | |
| --- | --- | --- | --- | --- | --- | --- | --- | --- |
| Covariates | B | SE | *t* | | | *p*-value | F (df) | R^2^ |
| Dependent variable: rCMglu of angular gyrus (df = 143) | | | | | | .009 | 3.549 (df = 4, 143) | .065 |
| Age (years) | -.003 | .001 | -1.825 | | | .070 |  |  |
| Sex | .024 | .024 | .989 | | | .324 |  |  |
| APOE ε4 genotype | -.073 | .031 | -2.392 | | | .018 |  |  |
| Serum TSH level | -.012 | .007 | -1.609 | | | .110 |  |  |
| Dependent variable: rCMglu of PCC (df = 143) | | | | | | .052 | 2.411 (df = 4, 143) | .063 |
| Age (years) | -.003 | .002 | | -1.796 | | .075 |  |  |
| Sex | -.024 | .025 | | -.928 | | .355 |  |  |
| APOE ε4 genotype | -.038 | .032 | | -1.201 | | .232 |  |  |
| Serum TSH level | -.012 | .008 | | -1.529 | | .129 |  |  |
| Dependent variable: rCMglu of precuneus (df = 143) | | | | | | .005 | 3.924 (df = 4, 143) | .074 |
| Age (years) | -.001 | .002 | | -.415 | | .679 |  |  |
| Sex | -.017 | .025 | | -.684 | | .495 |  |  |
| APOE ε4 genotype | -.081 | .032 | | -2.533 | | .012 |  |  |
| Serum TSH level | -.020 | .008 | | -2.584 | | .011 |  |  |
| Dependent variable : rCMglu in inferior temporal gyrus (df = 143) | | | | | | .152 | 1.705 (df = 4, 143) | .019 |
| Age (years) | -.003 | .001 | | | -2.217 | .028 |  |  |
| Sex | .024 | .019 | | | 1.239 | .217 |  |  |
| APOE ε4 genotype | -.024 | .024 | | | -.970 | .334 |  |  |
| Serum TSH level | -.001 | .006 | | | -.188 | .851 |  |  |
| Abbreviations: rCMglu, regional cerebral glucose metabolism; B, regression coefficient; SE, standard error; *APOE*, apolipoprotein E; PCC, posterior cingulate cortex; Multiple linear regression analysis was done for investigating the relationship between serum TSH and rCMglu after controlling age, gender, and *APOE* ε4 genotype (df for F statistics = 4,143). | | | | | | | | |
